# Latent *Mycobacterium tuberculosis* Infection Is Associated With a Higher Frequency of Mucosal-Associated Invariant T and Invariant Natural Killer T Cells

**DOI:** 10.3389/fimmu.2018.01394

**Published:** 2018-06-19

**Authors:** Dominic Paquin-Proulx, Priscilla R. Costa, Cassia G. Terrassani Silveira, Mariana P. Marmorato, Natalia B. Cerqueira, Matthew S. Sutton, Shelby L. O’Connor, Karina I. Carvalho, Douglas F. Nixon, Esper G. Kallas

**Affiliations:** ^1^Department of Microbiology, Immunology and Tropical Medicine, The George Washington University, Washington, DC, United States; ^2^School of Medicine, University of São Paulo, São Paulo, Brazil; ^3^Department of Pathology and Laboratory Medicine, University of Wisconsin-Madison, Madison, WI, United States; ^4^Hospital Israelita Albert Einstein, Instituto Israelita de Ensino e Pesquisa, São Paulo, Brazil

**Keywords:** mucosal-associated invariant T cells, invariant natural killer T cells, *Mycobacterium tuberculosis*, HIV-1, CCR6

## Abstract

Increasing drug resistance and the lack of an effective vaccine are the main factors contributing to *Mycobacterium tuberculosis* (Mtb) being a major cause of death globally. Despite intensive research efforts, it is not well understood why some individuals control Mtb infection and some others develop active disease. HIV-1 infection is associated with an increased incidence of active tuberculosis, even in virally suppressed individuals. Mucosal-associated invariant T (MAIT) and invariant natural killer T (iNKT) cells are innate T cells that can recognize Mtb-infected cells. Contradicting results regarding the frequency of MAIT cells in latent Mtb infection have been reported. In this confirmatory study, we investigated the frequency, phenotype, and IFNγ production of MAIT and iNKT cells in subjects with latent or active Mtb infection. We found that the frequency of both cell types was increased in subjects with latent Mtb infection compared with uninfected individuals or subjects with active infection. We found no change in the expression of HLA-DR, PD-1, and CCR6, as well as the production of IFNγ by MAIT and iNKT cells, among subjects with latent Mtb infection or uninfected controls. The proportion of CD4− CD8+ MAIT cells in individuals with latent Mtb infection was, however, increased. HIV-1 infection was associated with a loss of MAIT and iNKT cells, and the residual cells had elevated expression of the exhaustion marker PD-1. Altogether, the results suggest a role for MAIT and iNKT cells in immunity against Mtb and show a deleterious impact of HIV-1 infection on those cells.

## Introduction

*Mycobacterium tuberculosis* (Mtb) infection is a major cause of death globally. Several factors contribute to this phenomenon, including increased drug resistance (1) and the absence of a highly effective vaccine (2). In the majority of Mtb-infected individuals, there are no clinical signs of tuberculosis (TB), and the infection will be eliminated or remain latent (3). Immunocompromised individuals, including those infected with HIV-1, have a high risk of developing active Mtb infection. CD4+ and CD8+ T cells are believed to be important for immune control against Mtb (4, 5), but there is no known biomarker that can predict the progression from latent to active TB.

Innate-like unconventional T cells can rapidly produce cytokine after antigen exposure, and they have been implicated in the defense against Mtb (6). Mucosal-associated invariant T (MAIT) cells recognize pyrimidine intermediates derived from the riboflavin biosynthesis pathway (7, 8) presented by MR1 (9). Invariant natural killer T (iNKT) cells recognize glycolipids presented by CD1d (10, 11). Both MAIT and iNKT cells have been shown to directly recognize Mtb-infected cells (12, 13), and numerous studies have shown that their frequency is reduced in blood during active Mtb infection (12, 14–20). In humans, there is indirect evidence that MAIT and iNKT cells could play a role in controlling Mtb infection. For example, MAIT cells from tuberculous pleural effusions were shown to produce more IFNγ, IL-17, and granzyme B after stimulation with Mtb antigens (21). Both MAIT and iNKT cells are depleted during infection with HIV-1 (22–24) and HTLV-1 (25, 26). Infection with both of these pathogens is associated with a greater risk of developing active Mtb infection (27–30).

Results from non-human primate (NHP) animal models of Mtb infection suggest that CD8+ iNKT cells can play a protective role in preventing Mtb pathology (31), and MAIT cells were activated following BCG vaccination and Mtb infection (32). In mouse models, both iNKT and MAIT cells reduced bacterial burden following Mtb infection (13, 33). However, limited knowledge is available on the role of MAIT and iNKT cells in controlling Mtb infection in humans. Contradicting results have been reported regarding MAIT cell frequency in blood during latent Mtb infection (12, 17), and only one study has measured the frequency of iNKT cells in latent Mtb infection (16). A more detailed characterization of MAIT and iNKT cells in latent Mtb infection is still needed to better understand their role in immunological control of Mtb.

In the current confirmatory study, we evaluated MAIT and iNKT cell frequency, phenotype, and functionality in uninfected individuals and subjects with latent or active Mtb infection with and without HIV-1 infection. We found that both cell types were increased in subjects with latent Mtb infection compared with uninfected individuals and subjects with active Mtb infection. Latent Mtb infection was further associated with an increase in the proportion of CD4− CD8+ MAIT cells. Active Mtb infection was associated with elevated surface expression of the activation marker HLA-DR on both MAIT and iNKT cells, as well as of the exhaustion marker PD-1 on iNKT cells. No significant difference was observed between the groups in the production of IFNγ following *in vitro* stimulation of MAIT and iNKT cells.

## Materials and Methods

### Ethics Statement

HIV-1-uninfected (*n* = 41, age range 23–70) (Table 1) and -infected (*n* = 16, age range 22–68) (Table 2) subjects were enrolled in the study. There was no significant difference in age between any of the subgroups. Definition of Mtb infection was based on a positive PPD skin reaction above 10 mm and/or a positive TB-Spot test, in the absence (latent) or presence (active) of clinical signs or symptoms of TB. The study was approved by the University of São Paulo institutional review board (CAPPesq), and written informed consent was provided by all participants according to the Declaration of Helsinki. All samples were anonymized.

**Table 1:** Demographics and *Mycobacterium tuberculosis* (Mtb) status of HIV-1-uninfected subjects.

Subject	Gender	Mtb status
1	F	Latent
2	M	Uninfected
3	F	Uninfected
4	F	Uninfected
5	F	Uninfected
6	M	Latent
7	M	Latent
8	F	Latent
9	F	Uninfected
10	F	Uninfected
11	M	Uninfected
12	F	Latent
13	M	Latent
14	M	Uninfected
15	F	Uninfected
16	M	Latent
17	M	Uninfected
18	F	Latent
19	M	Active
20	F	Uninfected
21	M	Uninfected
22	F	Uninfected
23	F	Uninfected
24	F	Uninfected
25	F	Uninfected
26	F	Uninfected
27	F	Uninfected
28	F	Uninfected
29	M	Active
30	M	Active
31	M	Active
32	M	Active
33	M	Active
34	M	Active
35	M	Active
36	M	Active
37	F	Active
38	F	Latent
39	M	Active
40	M	Active
41	M	Active

**Table 2:** Demographics and *Mycobacterium tuberculosis* (Mtb) status of HIV-1-infected subjects.

Subject	Gender	Mtb status	Years on antiretroviral treatment
HIV-1	M	Latent	15
HIV-2	F	Uninfected	20
HIV-3	M	Uninfected	10
HIV-4	M	Latent	12
HIV-5	F	Uninfected	16
HIV-6	M	Latent	18
HIV-7	F	Latent	20
HIV-8	M	Latent	0
HIV-9	M	Latent	0.3
HIV-10	M	Uninfected	0.7
HIV-11	M	Uninfected	2
HIV-12	M	Uninfected	4
HIV-13	M	Uninfected	5
HIV-14	M	Uninfected	6
HIV-15	M	Uninfected	3
HIV-16	M	Uninfected	1

### Sample Collection

Peripheral blood mononuclear cells (PBMCs) were isolated by density-gradient sedimentation using Ficoll-Paque (Lymphoprep, Nycomed Pharma, Oslo, Norway). Isolated PBMCs were washed twice in Hank’s balanced salt solution (Gibco, Grand Island, NY, USA), and cryopreserved in RPMI 1640 (Gibco), supplemented with 20% heat inactivated fetal bovine serum (FBS; Hyclone Laboratories, Logan, UT, USA), 50 U/ml of penicillin (Gibco), 50 µg/ml of streptomycin (Gibco), 10 mM glutamine (Gibco), and 7.5% dimethylsulfoxide (Sigma, St. Louis, MO, USA). Cryopreserved cells from all subjects were stored in liquid nitrogen until used in the assays.

### Flow Cytometry and Antibodies

Cryopreserved specimens were thawed and washed, and counts and viability were assessed using the Countess Automated Cell Counter system (Invitrogen, Carlsbad, CA, USA). Cells were washed and stained in Brilliant Violet Stain Buffer (BD Biosciences, San Jose, CA, USA) at room temperature for 15 min in 96-well V-bottom plates in the dark. Samples were then washed and fixed using Cytofix/Cytoperm (BD Biosciences) before flow cytometry data acquisition. Intracellular staining was performed in Perm/Wash Buffer (BD Biosciences). Monoclonal antibodies used in flow cytometry: CD3 AF700, CD3 PerCP-Cy5.5 (both clone UCHT1), CD4 BV605 (clone RPA-T4), CD8 BV711 (clone RPA-T8), CD161 BV421 (clone DX12), CCR6 BV786 (clone 11A9), HLA-DR APC (clone L243), IFNγ APC (clone B27), and PD-1 PE-Cy7 (clone EH12.1) were all from BD Biosciences, TCRα24 FITC (clone C15) and TCR Vβ11 PE (clone C21) were from Beckman Coulter (Indianapolis, IN, USA), and TCR Vα7.2 PercP-Cy5.5 (clone 3C10) was from BioLegend (San Diego, CA, USA). Live/dead aqua fixable cell stain was from Life Technologies (Eugene, OR, USA). Data were acquired on a BD LSRFortessa instrument (BD Biosciences) and analyzed using FlowJo Version 9.8.5 software (TreeStar, Ashland, OR, USA).

### Functional Assays

Mucosal-associated invariant T cell function was determined *in vitro* using paraformaldehyde-fixed *Escherichia coli* stimulation (One Shot Top10, Life Technology, multiplicity of exposure 10) in the presence of 1.25 µg/ml anti-CD28 mAb (clone L293, BD Biosciences) (34). *E. coli* was fixed for 5 min in 1% paraformaldehyde. PBMCs were further cultured for 24 h at 37°C/5% CO_2_ in RPMI medium supplemented with 10% FBS. Monensin (Golgi Stop, BD Biosciences) was added during the last 6 h of the stimulation. iNKT cell function was determined *in vitro* using α-GalCer (KRN7000, Enzo Life Science, Farmingdale, NY, USA) at 100 ng/ml. PBMCs were further cultured for 8 h at 37°C/5% CO_2_ in RPMI medium supplemented with 10% FBS. Monensin (Golgi Stop, BD Biosciences) was added during the last 6 h of the stimulation.

### Statistical Analysis

All statistical analyses were performed using Graph Pad Prism version 6.0 h for Mac OSX (GraphPad Software, La Jolla, CA, USA). Results were tested for normal distribution, and appropriate ANOVA or Kruskal–Wallis test was used for comparison between groups. Mann–Whitney *U*-test was used for comparison between HIV-1-uninfected and infected subjects. *p*-Values ≤ 0.05 were considered statistically significant.

## Results

### Increased Frequency of MAIT and iNKT Cells in Latent Mtb Infection

**Figure 1:**
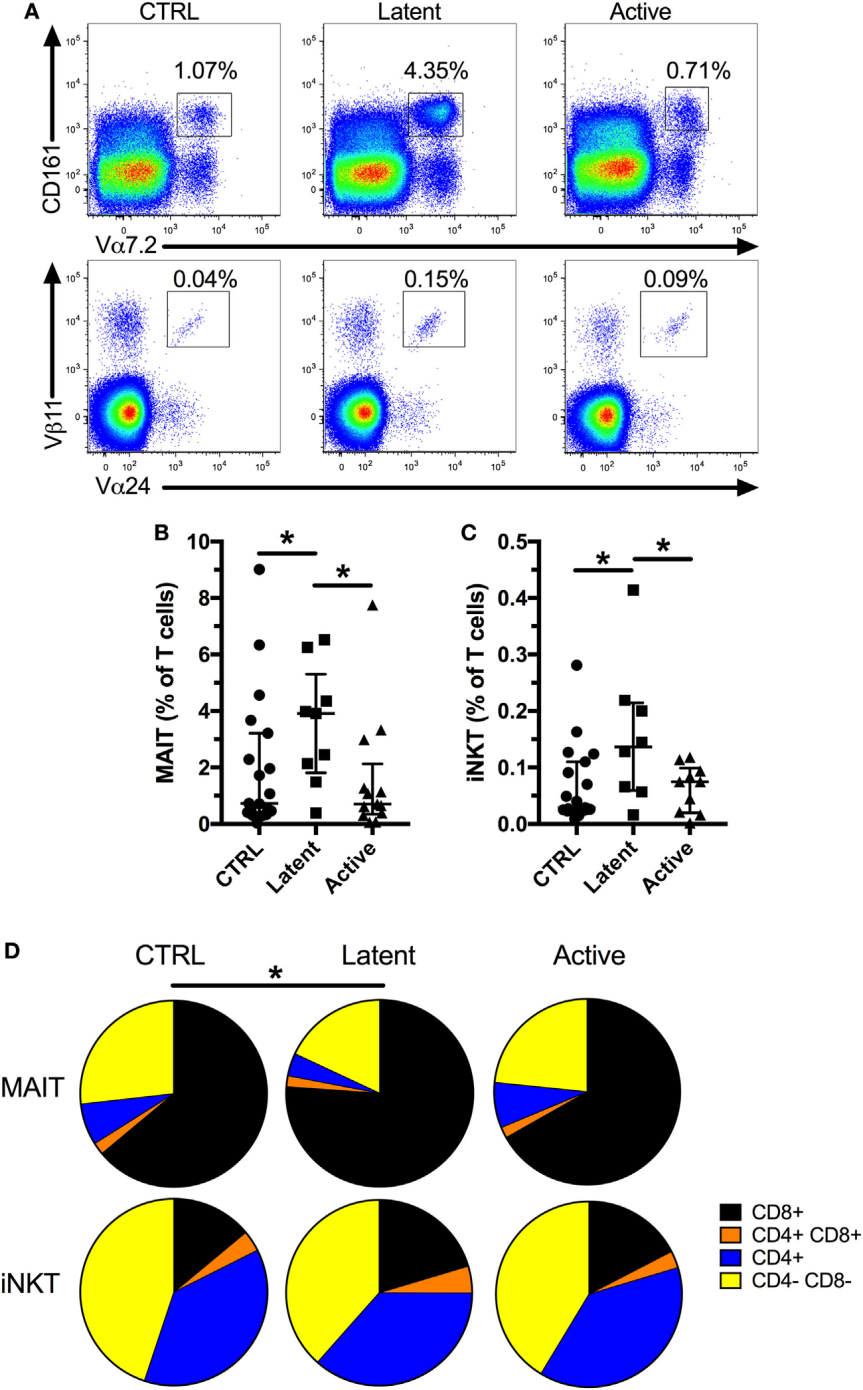
Mucosal-associated invariant T (MAIT) and invariant natural killer T (iNKT) cells are increased in subjects with latent *Mycobacterium tuberculosis* (Mtb infection). Representative flow plots showing MAIT and iNKT cell frequencies **(A)**. Frequency of MAIT cells in controls (*n* = 19), latent Mtb (*n* = 9), and subjects with active Mtb infection (*n* = 13) **(B)**. Frequency of iNKT cells in controls (*n* = 19), latent Mtb (*n* = 8), and subjects with active Mtb infection (*n* = 10) **(C)**. Proportion of CD8+, CD4+ CD8+, CD4+, and CD4− CD8− cells in MAIT (top row) and iNKT cell (bottom row) for controls (left column), latent Mtb (middle column), and subjects with active Mtb infection (right column) **(D)**. The lines and whiskers represent the median and interquartile range, respectively (*p < 0.05).

First, to confirm previous studies, we evaluated the frequency of MAIT and iNKT cells in a cohort of patients with latent (age range 23–63, median 39) or active Mtb infection (age range 26–58, median 45) compared with uninfected controls (Table 1; Figure 1A, age range 26–70, median 42). We found no difference in the frequency of both cell types between active Mtb infection and uninfected controls (Figures 1B,C). However, there was a significant increase in the frequency of MAIT and iNKT cells in individuals with latent Mtb infection compared with active Mtb infection, or uninfected controls. Next, we investigated if there was a change in the CD4+ and CD8+ subset distribution of MAIT and iNKT cells between the groups. As expected, MAIT cells were mostly CD4− CD8+ and CD4− CD8−, whereas iNKT cells were mostly CD4+ CD8− and CD4− CD8−, in all three groups. Furthermore, we found that there was an increase in the proportion of CD4− CD8+ MAIT cells in the latent Mtb infection group compared with the control group (Figure 1D). No change in the distribution of iNKT cell subsets was observed between the groups. Our results suggest that latent Mtb infection is associated with an increased MAIT and iNKT cell frequency.

### MAIT and iNKT Cells Are Activated in Active, but Not Latent, Mtb Infection

**Figure 2:**
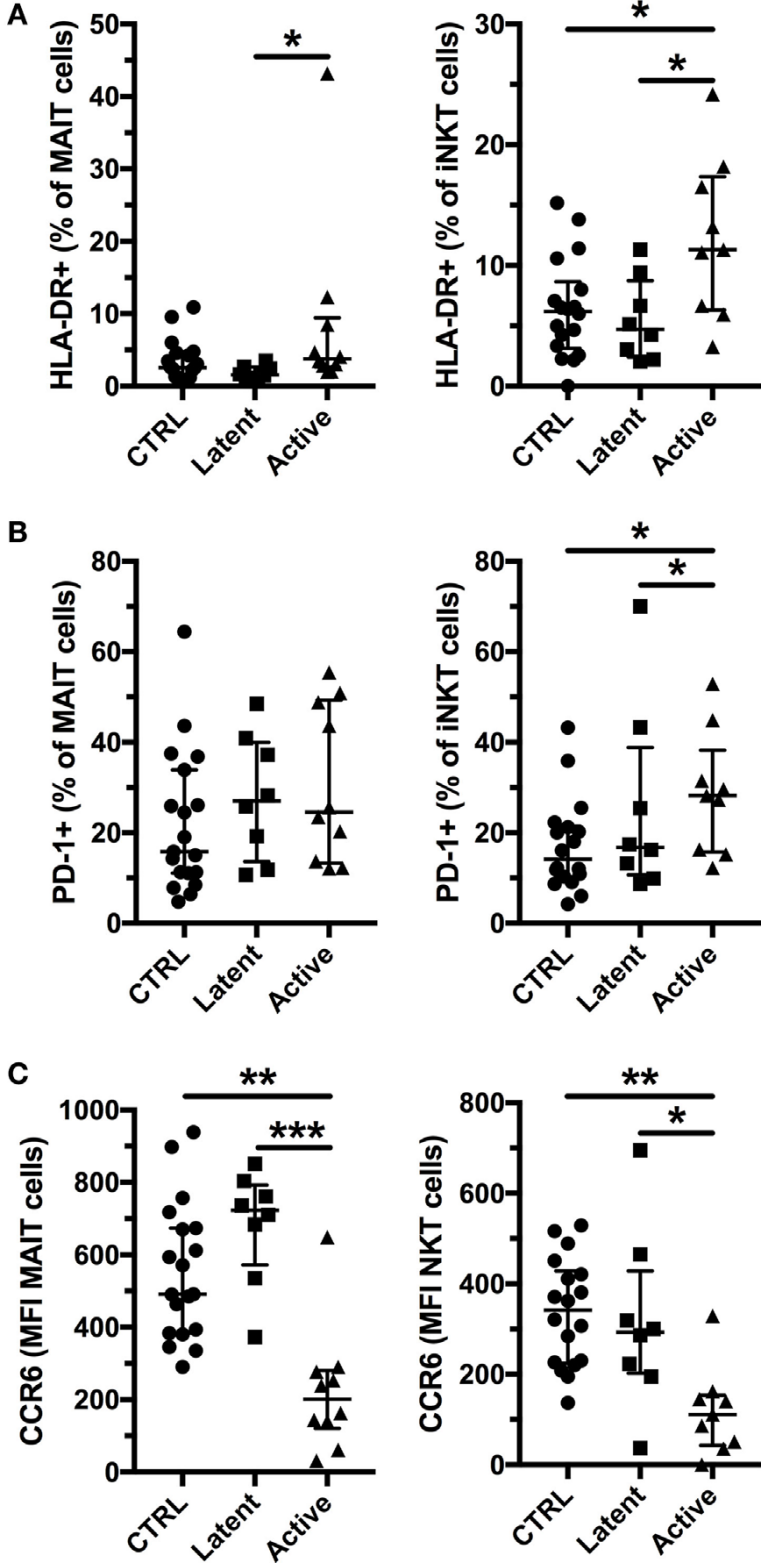
Invariant natural killer T (iNKT) cells express activation and exhaustion markers together with reduced CCR6 expression in active tuberculosis. Expression of HLA-DR by mucosal-associated invariant T (MAIT) and iNKT cells **(A)**, expression of PD-1 by MAIT and iNKT cells **(B)**, and levels of CCR6 expression by MAIT and iNKT cells **(C)** according to their *Mycobacterium tuberculosis* infection status (controls *n* = 19, latent *n* = 8, and active *n* = 10 for MAIT cells and *n* = 9 for iNKT cells). The lines and whiskers represent the median and interquartile range, respectively (*p < 0.05, **p < 0.01, and ***p < 0.001).

Previous studies have reported that MAIT and iNKT cells have increased expression of activation and exhaustion markers, but reduced expression of CCR6 in active Mtb infection (15, 16, 32, 35). Therefore, we investigated the expression of HLA-DR, PD-1, and CCR6 in latent, and active Mtb infection (Figure S1 in Supplementary Material). We found increased expression of HLA-DR on MAIT and iNKT cells in active, but not in latent, Mtb infection (Figure 2A; Figure S1A in Supplementary Material). Increased immune activation has been associated with a lower frequency of MAIT cells in chronic viral infections and in conditions associated with inflammation (23, 25, 36). In our cohort, there was no significant association between HLA-DR expression and the frequency of MAIT and iNKT cells in active Mtb infection (Figure S2A in Supplementary Material). Furthermore, PD-1 expression was elevated on iNKT cells, but not on MAIT cells, in active Mtb infection (Figure 2B; Figure S1B in Supplementary Material). CCR6 expression was reduced only in active Mtb infection for both MAIT and iNKT cells (Figure 2C; Figure S1C in Supplementary Material). Reduced CCR6 surface expression could be indicative of ligand binding and an early sign of tissue recruitment and activation. However, there was no significant association between CCR6 expression and the frequency of MAIT and iNKT cells or their HLA-DR expression in subjects with active Mtb infection (Figures S2B,C in Supplementary Material). Our results show that active Mtb infection is associated with increased activation and exhaustion of MAIT and iNKT cells compared with uninfected controls, in contrast to latent Mtb infection.

### Normal MAIT and iNKT Cell IFNγ Production in Latent and Active Mtb Infection

**Figure 3:**
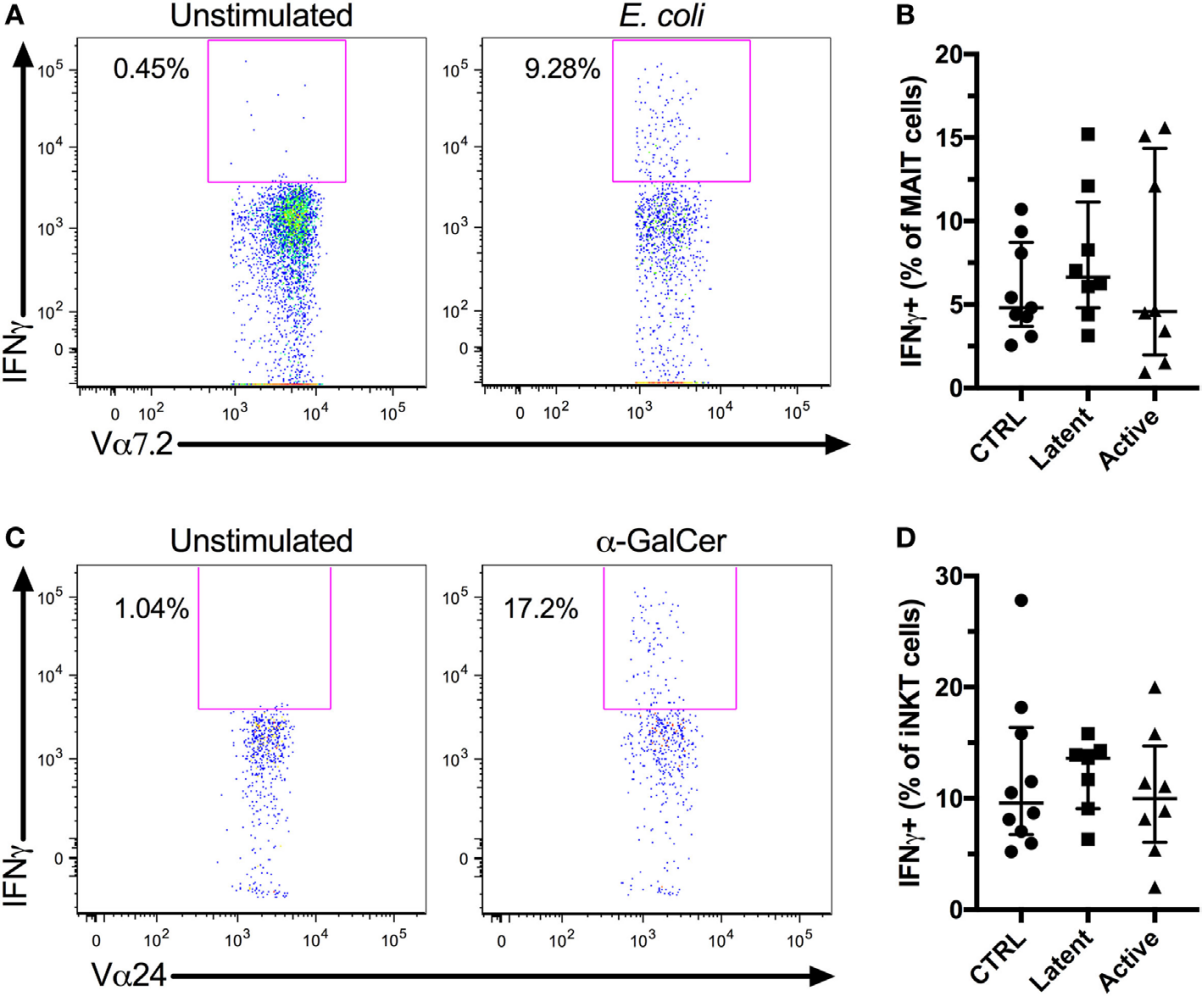
*In vitro* IFNγ production of mucosal-associated invariant T (MAIT) and invariant natural killer T (iNKT) cells. Peripheral blood mononuclear cells (PBMCs) from HIV-1-uninfected subjects were stimulated with *Escherichia coli* or α-GalCer, and IFNγ production was evaluated for MAIT and iNKT cell, respectively. Representative flow plots showing MAIT cells IFNγ production by unstimulated and *E. coli*-stimulated PBMCs **(A)**. IFNγ production by MAIT cells after *E. coli* stimulation for controls (*n* = 9), latent *Mycobacterium tuberculosis* (Mtb) (*n* = 8), and subjects with active Mtb infection (*n* = 8) **(B)**. Representative flow plots showing iNKT cells IFNγ production by unstimulated and α-GalCer-stimulated PBMCs **(C)**. IFNγ production by iNKT cells after α-GalCer stimulation for controls (*n* = 10), latent Mtb (*n* = 7), and subjects with active Mtb infection (*n* = 8) **(D)**.

The IFNγ response is essential in the control of Mtb (37). Furthermore, MAIT and iNKT cells have been shown to recognize Mtb-infected cells *in vitro* (12, 13). Thus, a high innate T cell IFNγ production could be associated with control of Mtb infection. We evaluated the IFNγ production by MAIT and iNKT cells *in vitro* following antigen stimulation. PBMCs were stimulated with fixed *E. coli* or α-GalCer to activate MAIT and iNKT cells, respectively, and IFNγ production was evaluated by flow cytometry (Figures 3A,C). There was a small, albeit non-significant, increase in the production of IFNγ by MAIT and iNKT cells in individuals with latent Mtb compared with uninfected controls and individuals with active Mtb infection. However, there was no significant change in the production of IFNγ between all groups for both cell types (Figures 3B,D). There was also no association between the age of the subjects and the production of IFNγ by the MAIT or iNKT cells (*r* = 0.1567, *p* = 0.4544 and *r* = −0.0930, *p* = 0.6585, respectively).

### Reduced MAIT and iNKT Cells in HIV-1 Infection Is Independent of Mtb Infection

**Figure 4:**
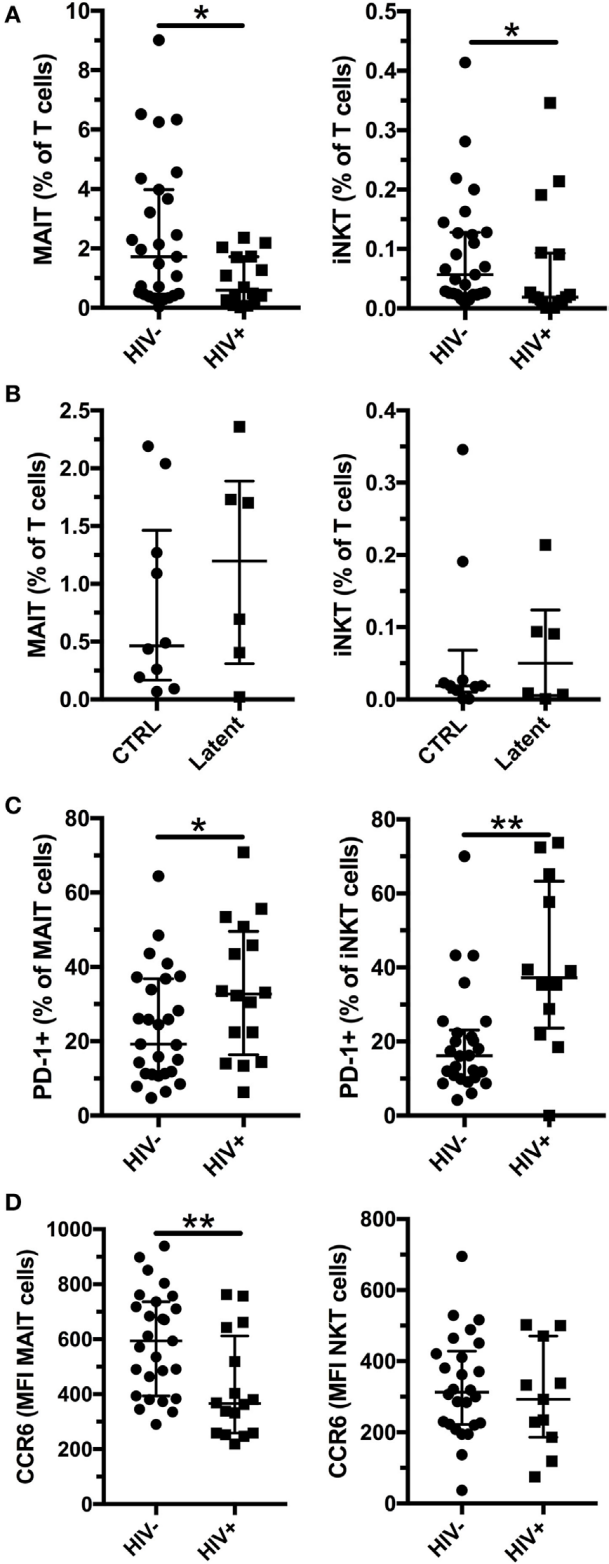
Mucosal-associated invariant T (MAIT) and invariant natural killer T (iNKT) cells are reduced in HIV-1 infection. Frequency of MAIT (left panel) and iNKT (right panel) cells in HIV-1-uninfected (*n* = 27) and infected (*n* = 16) subjects without active *Mycobacterium tuberculosis* (Mtb) infection **(A)**. Frequency of MAIT (left panel) and iNKT (right panel) cells in Mtb uninfected (*n* = 10) and with latent Mtb infection (*n* = 6) HIV-1-infected subjects **(B)**. PD-1 expression **(C)** and CCR6 expression **(D)** by MAIT (left panel) and iNKT (right panel) in HIV-1-uninfected (*n* = 27) and -infected (*n* = 16) subjects without active Mtb infection. The lines and whiskers represent the median and interquartile range, respectively (*p < 0.05 and **p < 0.01).

HIV-1 infection is associated with an increased susceptibility to Mtb infection and more severe TB disease, even in individuals on long-term antiretroviral treatment (ART) (29, 30). We enrolled HIV-1-infected subjects without active Mtb infection (Table 2, age range 31–68, median 41.5, for Mtb uninfected subjects and age range 22–52, median 43, for individuals with latent Mtb infection) and investigated their MAIT and iNKT cell frequency and phenotype. All but one of the HIV-1-infected individuals were on ART, and the median duration of treatment was 5.5 years. As previously reported, the frequencies of MAIT and iNKT cells were decreased in HIV-1-infected individuals without active Mtb infection compared with HIV-1-uninfected subjects (Figure 4A). Similar to HIV-1-uninfected subjects, there was a trend for increased frequencies of MAIT (median 0.46 vs 1.20%) and iNKT cells (median 0.02 vs 0.05%) in HIV-1-infected subjects with latent Mtb infection, compared with the Mtb-uninfected individuals (Figure 4B). This increase did not reach statistical significance likely due to the low number of subjects in each group. There was a positive association between the frequencies of MAIT and iNKT cells in uninfected and HIV-1-infected subjects (Figures S3A,B in Supplementary Material). PD-1 expression was increased on MAIT and iNKT cells in HIV-1-infected individuals (Figure 4C). Finally, CCR6 levels were reduced on MAIT cells in HIV-1 infection, but not on iNKT cells (Figure 4D). Our results indicate that alterations of MAIT and iNKT cells caused by HIV-1 infection are not restored after long-term ART.

## Discussion

Several populations of innate T cells have been proposed to play a role in the immune response against Mtb (6). In this study, we sought to confirm previous investigations of the frequency of MAIT and iNKT cells in latent and active Mtb infections (12, 16–18). We found an increase in their frequencies in latent Mtb infection. Our observation that there was a higher frequency of MAIT cells in latent Mtb infection is in agreement with the results of Gold et al. (12), but in contrast to those of Wong et al. (17). One difference between those studies was how MAIT cells were defined. Wong et al. identified MAIT cells as CD161++ CD8+ T cells whereas Gold et al. identified MAIT cells as CD8+ Vα7.2+ T cells. In our studies, we used CD161++ Vα7.2+ T cells to identify MAIT cells, which includes the CD8− subset of MAIT cells. Latent Mtb infection could induce proliferation of MAIT and iNKT cells by constant low exposure to stimulating antigens. We would expect this to be associated with higher expression of activation markers, but we found no change in HLA-DR expression on MAIT and iNKT cells in latent Mtb infection. Furthermore, NHP studies have shown no change in peripheral MAIT cell frequency following BCG vaccination or Mtb infection (32). Another possibility is that a higher frequency of innate T cells before infection is associated with control of Mtb. Addressing these would require a longitudinal study.

Interestingly, we found that in latent Mtb infection there was an increase in the proportion of CD4− CD8+ MAIT cells but no change in iNKT cell subsets. There is growing interest in understanding the heterogeneity of MAIT cells (38–40). CD8+ MAIT cells have a higher expression of CCR6 compared with the CD8− CD4− subset, and they have higher levels of Granzyme A, Granzyme K, and Perforin compared with the CD4+ subset (38). Thus, CD8+ MAIT cells could have a higher combined capacity to migrate to and kill Mtb-infected cells compared with the other subsets of MAIT cells.

We found that active Mtb infection was associated with increased expression of HLA-DR and decreased expression of CCR6 on MAIT and iNKT cells. Lower CCR6 levels could impair the capacity of the cells to migrate to the lungs. We also found increased PD-1 expression on iNKT cells, but only in active Mtb infection. Notably, there was no change in the IFNγ production of both cell types after antigen stimulation in active Mtb infection. This is in contrast with a previous study that found less IFNγ production from MAIT cells in active Mtb infection (18). It remains to be determined if elevated HLA-DR and PD-1 expression by MAIT and iNKT cells in active Mtb infection are a cause or a consequence of the disease. In this regard, PD-1 expression by conventional CD4+ T cells is needed to prevent immune mediated pathology in response to Mtb infection (41). Chronic activation of MAIT and iNKT cells by direct recognition of Mtb-infected cells and exposure to elevated levels of inflammatory cytokines could lead to this abnormal phenotype.

*Mycobacterium tuberculosis* infection is a major comorbidity associated with HIV-1 infection, and HIV-1-infected subjects on ART remain at higher risk of developing active Mtb infection (29, 30). We report here a concomitant decline in MAIT and iNKT cells in a cohort of mostly ART-treated HIV-1-infected individuals. Residual MAIT and iNKT cells had elevated levels of PD-1, a marker associated with exhaustion. Furthermore, residual MAIT cells had lower levels of CCR6, suggesting an impaired capacity to migrate to mucosal tissue. This dysregulation of MAIT and iNKT cells was observed in some HIV-1-infected subjects who were on ART for over 15 years, suggesting that these cells do not recover even with viral suppression. Combination of ART with immunotherapies such as IL-7 and IL-2 has shown some capacity to increase MAIT and iNKT cell frequency (42, 43). Altogether, our results suggest that HIV-1-associated defects in MAIT and iNKT cells could be in part responsible for the increased susceptibility of HIV-1-infected individuals to Mtb infection and more severe disease progression.

One limitation of our study is that we only explored cell population in the peripheral blood. Studying MAIT and iNKT cells in the lung would be highly relevant in the context of Mtb infection. Studies using animal models may be better positioned to address this question.

Overall, our results suggest a role for MAIT and iNKT cells in the successful immune control of Mtb infection. Further research is needed to develop strategies to restore these cells in ART-treated HIV-1-infected individuals.

## Ethics Statement

The study was approved by the University of São Paulo institutional review board (CAPPesq), and written informed consent was provided by all participants according to the Declaration of Helsinki. All samples were anonymized.

## Author Contributions

Performed experiments: DP-P, PC, CS, MM, NC, and MS. Analyzed data: DP-P. Design study: DP-P, SO, KC, DN, and EK. Wrote the manuscript: DP-P, DN, and EK. All the authors reviewed the manuscript.

## Conflict of Interest Statement

The authors declare that the research was conducted in the absence of any commercial or financial relationships that could be construed as a potential conflict of interest. The reviewer SJ and handling Editor declared their shared affiliation.

## Abbreviations

Mtb, *Mycobacterium tuberculosis*; MAIT, mucosal-associated invariant T; iNKT, invariant natural killer T; NHP, non-human primate; PBMC, peripheral blood mononuclear cell; ART, antiretroviral treatment.
