# Supplementary material for: Latent Mycobacterium tuberculosis Infection Is Associated With a Higher Frequency of Mucosal-Associated Invariant T and Invariant Natural Killer T Cells
Source: Front Immunol. 2018 Jun 19;9:1394. doi: 10.3389/fimmu.2018.01394 (PMC6018487; doi:10.3389/fimmu.2018.01394)
Supplement: Supplementary file 1 [file data_sheet_1.docx]

**Sup. Figure 1**


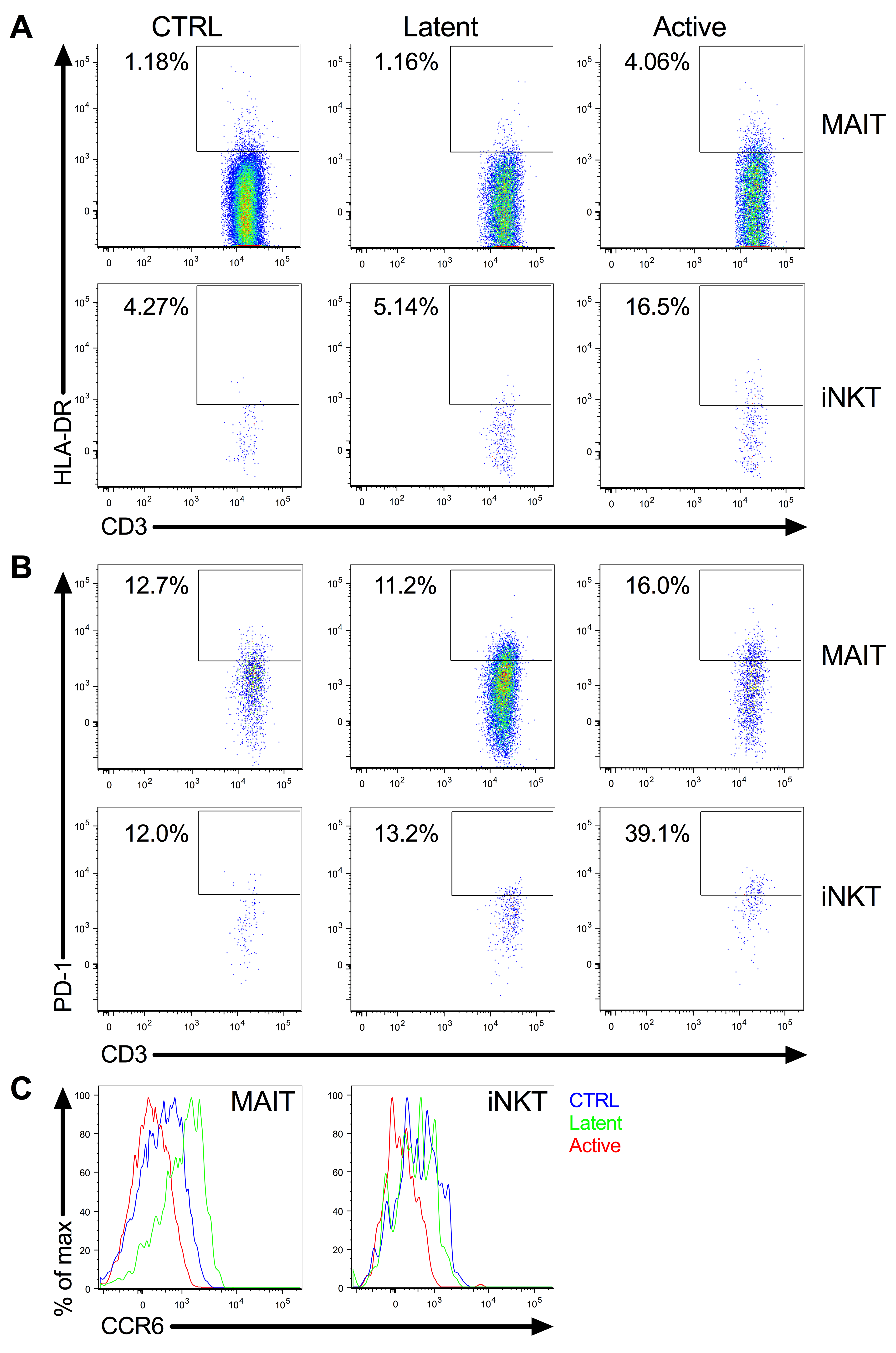


**Sup. Figure 1.** Representative flow plots showing HLA-DR, PD-1 and CCR6 expression on MAIT and iNKT cells.

**Sup. Figure 2.**


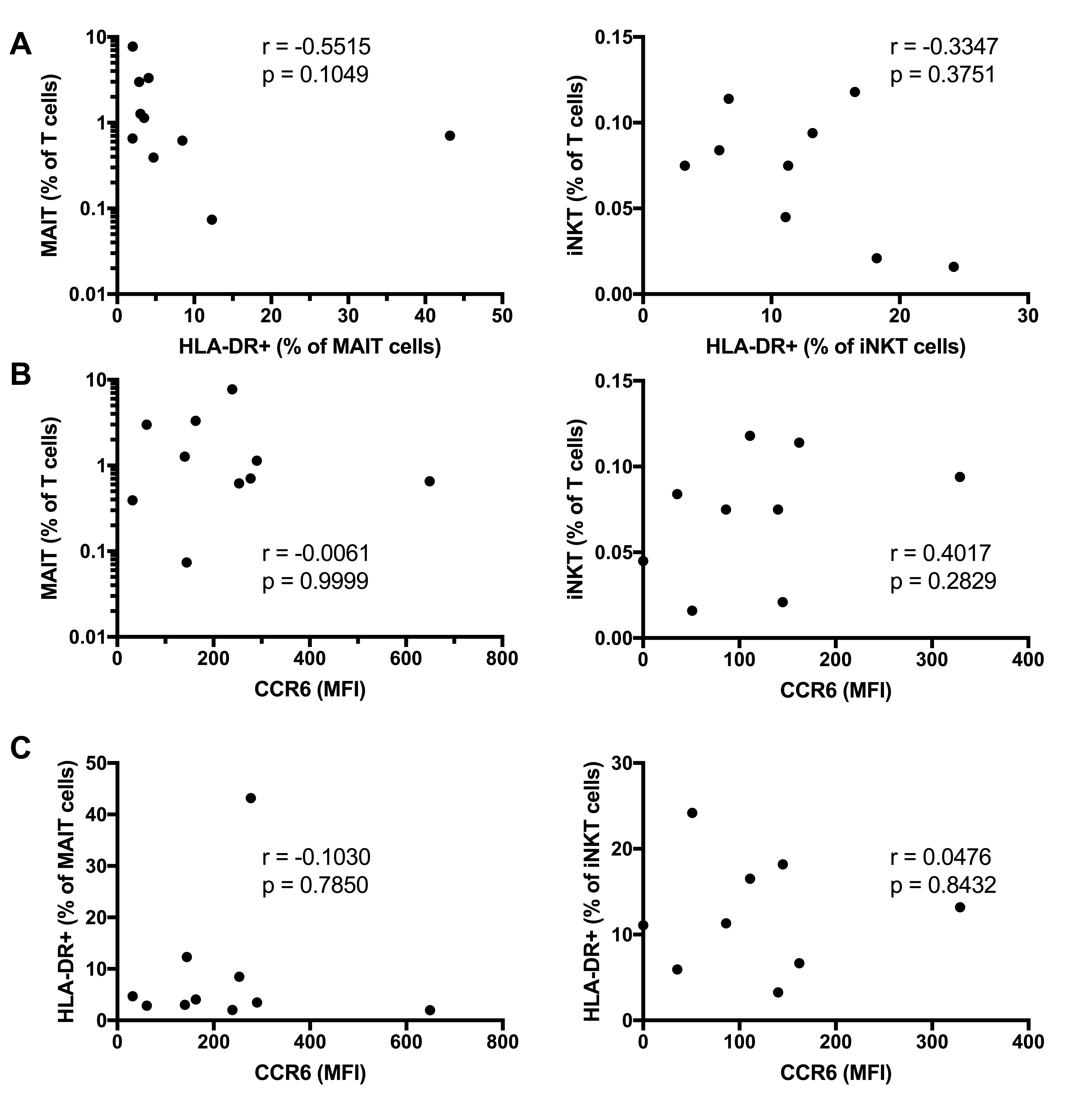


**Sup. Figure 2.** Associations between HLA-DR expression and frequency of MAIT (left panel, n= 10) and iNKT (right panel, n= 9) cells in active Mtb infection (A). Associations between CCR6 expression and frequency of MAIT (left panel, n= 10) and iNKT (right panel, n= 9) cells in active Mtb infection (B). Associations between CCR6 expression and HLA-DR expression by MAIT (left panel, n= 10) and iNKT (right panel, n= 9) cells in active Mtb infection (C).

**Sup. Figure 3.**


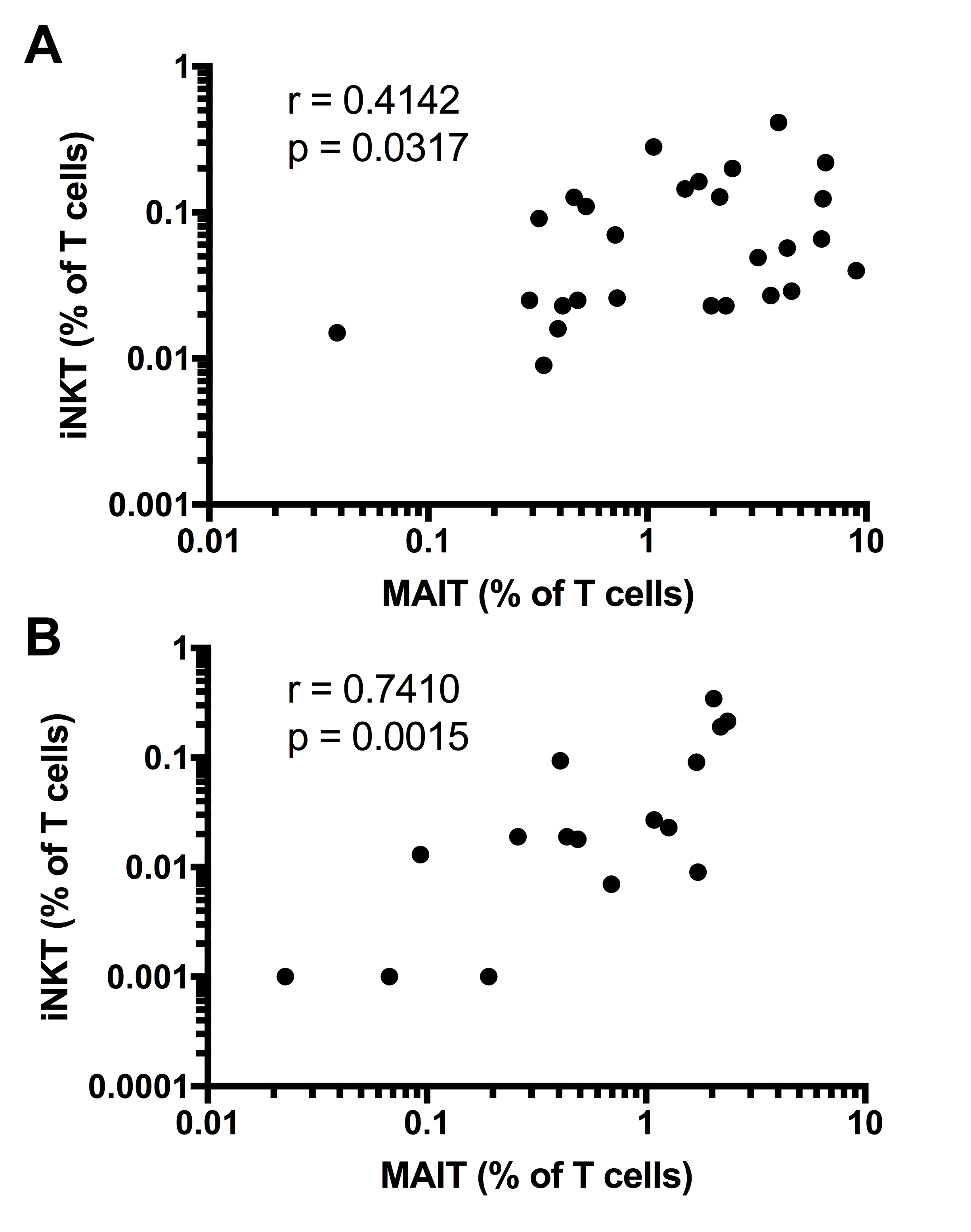


**Sup. Figure 3.** Associations between frequency of MAIT cells and iNKT cells in HIV-uninfected (n= 27, A) and HIV-infected (n = 16, B) subjects without active Mtb infection.
